# Complete genome sequences of seven *Polynucleobacter* sp. subcluster PnecB2 strains (HIN5, HIN6, HIN7, HIN8, HIN9, HIN10, and HIN11) isolated from a shallow brackish eutrophic lake in Japan

**DOI:** 10.1128/mra.01402-25

**Published:** 2026-03-06

**Authors:** Hiroaki Masuoka, Yusuke Ogata, Keiji Watanabe, Rina Kurokawa, Shusuke Takemine, Misa Takagi, Chie Shindo, Wataru Suda

**Affiliations:** 1 RIKEN Center for Integrative Medical Sciences 198286, Yokohama, Kanagawa, Japan; 2 Center for Environmental Science in Saitama 290710, Kazo, Saitama, Japan; Montana State University, Bozeman, Montana, USA

**Keywords:** whole genome shotgun, *Polynucleobacter*subcluster PnecB2

## Abstract

The *Polynucleobacter* subcluster PnecB comprises bacteria representing a ubiquitous taxon of freshwater bacterioplankton. Here, we report the complete genome sequences of seven *Polynucleobacter* (PnecB2) strains, HIN5, HIN6, HIN7, HIN8, HIN9, HIN10, and HIN11, isolated from the surface water of a shallow, brackish, eutrophic lake in Japan.

## ANNOUNCEMENT

Free-living freshwater bacterioplankton of the genus *Polynucleobacter* belonging to subcluster PnecB are distributed worldwide and occasionally dominant (1–4). To date, only two species (*Polynucleobacter acidiphobus* and *Polynucleobacter difficilis*) have been described (5, 6). Here, we report the complete genome sequences of seven *Polynucleobacter* spp. subcluster PnecB2 strains: HIN5 (JCM 18220), HIN6 (JCM 36265), HIN7 (JCM 36266), HIN8 (JCM 36267), HIN9 (JCM 36268), HIN10 (JCM 36269), and HIN11 (JCM 36270) isolated from lake Hinuma, a shallow brackish eutrophic lake in Ibaraki, Japan (36°16′28.8″N 140°28′43.2″E). A surface water sample (0–50-cm depth) was collected on 21 July 2010 and filtered through a glass-fiber filter with 0.7-µm particle retention capacity (Whatman). The filtrate was spread onto modified Reasoner’s 2A (MR2A) agar and incubated at 25°C for 7 days (7). Colonies were subcultured in the MR2A liquid medium (pH 7.2), incubated under the same conditions with shaking, and preserved at −80°C in MR2A with 20% glycerol. Genomic DNA was extracted from cultures revived on glycerol stocks. The isolates were identified as members of the genus *Polynucleobacter* based on the 16S rRNA gene sequence analysis, showing high similarity to *Polynucleobacter* strains of the PnecB cluster in BLAST and EzBioCloud (8) searches.

**Fig 1:**
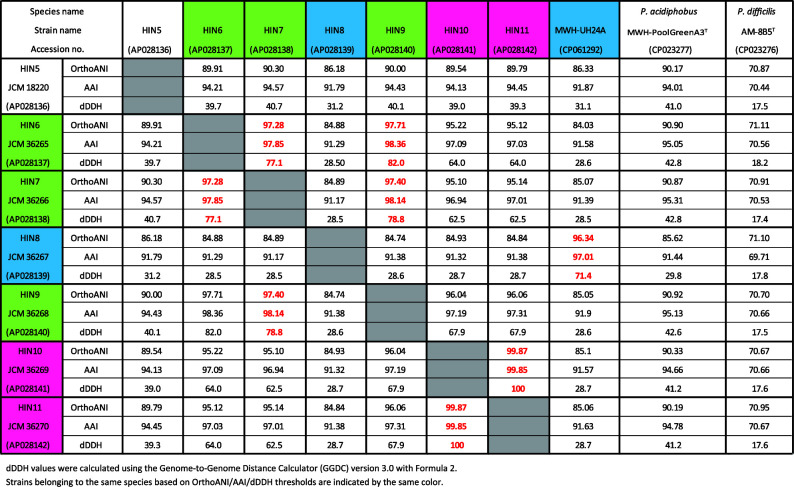
OrthoANI, AAI, and dDDH values among strains HIN5, HIN6, HIN7, HIN8, HIN9, HIN10, HIN11, MWH-UH24A, and two *Polynucleobacter* subcluster PnecB type strains.

Genomic DNA was extracted using enzymatic lysis and phenol-chloroform-isoamyl alcohol method (9). Whole-genome sequencing was performed using the Sequel II System (PacBio). Genomic DNA was sheared using a g-TUBE (Covaris), and libraries were prepared with the SMRTbell Express Template Preparation Kit v2.0 (PacBio) without size selection. The PacBio reads were converted to HiFi reads using CCS software v6.2.0. All seven strains were assembled using the assembler Canu v2.1.1 (10) with the parameters --pacbio-hifi, minReadLength = 2200, and minOverlapLength = 2200. Contigs with low read depth (<5) were eliminated. Contigs were remapped with Minimap2 v2.24-r1122 for circularization; terminal overlaps were detected from self-alignments and trimmed iteratively. The largest circular contig was determined as the chromosome contig. The quality of the genome assemblies for strains HIN5 through HIN11 was assessed using CheckM v1.2.2 (11). The assemblies showed completeness values ranging from 93.30 to 93.72%. Contamination was 0.2% for all strains, except for HIN8, which showed 0.4%. Strain heterogeneity was 0% for all genomes. The genomes were annotated and rotated to start from the *dnaA* gene using DFAST v1.2.20 (12). Default parameters were used for all software analyses unless otherwise specified. The obtained reads and genome assemblies are summarized in Table 1, which also includes key assembly statistics (e.g., contig number and genome size). Average nucleotide identity by orthology (OrthoANI) was calculated using OAT (13); average amino acid identity (AAI) values were calculated using the AAI calculator provided by the enveomics collection of tools (14); and digital DNA-DNA hybridization (dDDH) values were calculated using the Genome-to-Genome Distance Calculator (GGDC) version 3.0 with Formula 2 (15). Comparative values for strains HIN6, HIN7, HIN8, HIN9, HIN10, HIN11, and MWH-UH24A, together with two *Polynucleobacter* subcluster PnecB type strains, are shown in Fig. 1. Phylogenetic analysis based on 120 conserved marker genes placed all seven strains within the PnecB2 subcluster.

**TABLE 1:** Summary of reads and contigs data for *Polynucleobacter* sp. (PnecB) strains HIN5, HIN6, HIN7, HIN8, HIN9, HIN10, and HIN11

Parameter	Data for strain						
	HIN5	HIN6	HIN7	HIN8	HIN9	HIN10	HIN11
Data for quality-checked Sequel reads							
No. of reads	24,138	8,576	11,551	30,000	13,724	14,173	10,297
Total no. of bases	321,221,469	117,642,566	159,795,315	394,158,881	188,374,284	194,528,725	142,568,605
*N*_50_ (bp)	13,096	13,543	13,679	12,931	13,574	13,586	13,687
Total no. of contigs	1	1	1	1	1	1	1
Number of CDSs^*a*^	1,850	1,794	1,781	2,012	1,782	1,777	1,774
Number of rRNAs	3	3	3	3	3	3	3
Number of tRNAs	38	39	38	40	39	39	39
Coverage (×)	171	32	44	95	51	53	39
BioProject accession no.	PRJDB15919	PRJDB15919	PRJDB15919	PRJDB15919	PRJDB15919	PRJDB15919	PRJDB15919
BioSample accession no.	SAMD00613833	SAMD00613834	SAMD00613835	SAMD00613836	SAMD00613837	SAMD00613838	SAMD00613839
Sequence Read Archive (SRA) accession no.	DRR472213	DRR472214	DRR472215	DRR472216	DRR472217	DRR472218	DRR472219
Chromosome data							
Genome size (bp)	1,880,609	1,850,487	1,795,157	1,984,719	1,809,006	1,813,778	1,813,265
GC content (%)	47.8	48.0	48.1	46.5	48.0	48.0	48.0
GenBank/ENA/DDBJ accession no.	AP028136	AP028137	AP028138	AP028139	AP028140	AP028141	AP028142

^*a*^ CDSs, coding DNA sequences.

## Data Availability

The genome sequences and raw sequencing data are available at GenBank/ENA/DDBJ under BioProject accession number PRJDB15919. Information on the BioSample, DDBJ Sequence Read Archive (DRA), and DDBJ accession numbers is provided in Table 1.
